# Spatiotemporal Analysis of Complex Emission Dynamics in Port Areas Using High-Density Air Sensor Network

**DOI:** 10.3390/toxics12100760

**Published:** 2024-10-19

**Authors:** Jun Pan, Ying Wang, Xiaoliang Qin, Nirmal Kumar Gali, Qingyan Fu, Zhi Ning

**Affiliations:** 1 Shanghai Environmental Monitoring Center, Shanghai 200233, China; semcpan@126.com; 2 Sapiens Environmental Technology Co., Ltd., Dongguan 523690, China; wangying@sapiens-envtech.com; 3 Division of Environment and Sustainability, The Hong Kong University of Science and Technology, Hong Kong 999077, China; xqinaf@connect.ust.hk (X.Q.); nkgali@ust.hk (N.K.G.); 4 State Environmental Protection Key Laboratory of Formation and Prevention of Urban Air Pollution Complex, Shanghai Academy of Environmental Sciences, Shanghai 200233, China

**Keywords:** cargo terminals, lowest percentile method, nitric oxide, maritime shipping emissions

## Abstract

Cargo terminals, as pivotal hubs of mechanical activities, maritime shipping, and land transportation, are significant sources of air pollutants, exhibiting considerable spatiotemporal heterogeneity due to the complex and irregular nature of emissions. This study employed a high-density air sensor network with 17 sites across four functional zones in two Shanghai cargo terminals to monitor NO and NO_2_ concentrations with high spatiotemporal resolution post sensor data validation against regulatory monitoring stations. Notably, NO and NO_2_ concentrations within the terminal surged during the night, peaking at 06:00 h, likely due to local regulations on heavy-duty diesel trucks. Spatial analysis revealed the highest NO concentrations in the core operational areas and adjacent roads, with significantly lower levels in the outer ring, indicating strong emission sources and limited dispersion. Employing the lowest percentile method for baseline extraction from high-resolution data, this study identified local emissions as the primary source of NO, constituting over 80% of total emissions. Elevated background concentrations of NO_2_ suggested a gradual oxidation of NO into NO_2_, with local emissions contributing to 32–70% of the total NO_2_ concentration. These findings provide valuable insights into the NO and NO_2_ emission characteristics across different terminal areas, aiding decision-makers in developing targeted emission control policies.

## 1. Introduction

Cargo ports play a crucial role in the growth of international trade and commerce [1,2,3]. However, the development, scale, and activities of ports have led to significant environmental concerns, particularly regarding air quality in and around these logistical hubs [4,5,6]. Nitrogen oxides (NOx) are one of the main air pollutants within ports, with increased emissions primarily attributed to the use of high-emission fuels, the intensity of terminal operations, and the lack of effective control measures [7,8]. Heavy-duty diesel trucks, the primary freight vehicles in land transportation associated with terminal activities, exhibit high-emission characteristics, and the relatively poor dispersion conditions within the terminals may further exacerbate pollutant concentrations. The emissions from ships within the port facility, both during their entry and exit and from onboard power generation, contribute to NOx levels, posing potential hazards to the environment and human health in terminal areas and nearby coastal cities. These hazards result in deteriorating air quality, including the formation of secondary pollutants, which subsequently lead to various health issues, particularly respiratory diseases [9,10,11].

Several studies have explored air pollution issues in terminal areas, demonstrating that pollutant concentrations increase within a few hundred meters of vehicular-related emission sources, with heavy-duty diesel trucks servicing terminal facilities significantly contributing to the increased pollutant concentration [12,13,14]. Methods like the AERMOD model and Emission Ratio–Positive Matrix Factorization (ER-PMF) have been employed to assess and apportion emissions in terminals, highlighting the substantial impact of both exhaust and non-exhaust sources [15,16,17]. However, existing studies often focus on coastal terminal areas, neglecting inland terminals, and sometimes rely on data from a single monitoring site, failing to capture the comprehensive spatial and temporal heterogeneity of pollutants [18,19,20]. Traditional pollutant monitoring in ports, primarily through fixed air quality monitoring stations, faces challenges due to high costs and limited spatial coverage. The advent of low-cost sensor (LCS) networks presents a viable solution to these challenges, offering flexible, extensive, and high-resolution monitoring capabilities [21,22,23,24]. Studies like those of Dimitriou et al. [25] and Frederickson et al. [26] have demonstrated the efficacy of LCS networks in capturing the spatial variability of pollutants like PM_2.5_ and providing insights into pollution patterns and sources. Furthermore, the impact of port emissions on local air quality and health has been increasingly scrutinized. Studies have identified the direct influence of ship and truck emissions on the concentration of pollutants like PM_2.5_ and NOx, as well as their subsequent health impacts in port cities [27,28]. Strategies to reduce these emissions, such as the establishment of Sulphur Emission Control Areas (SECAs), have been shown to potentially reduce concentrations of key pollutants and improve local air quality [29].

In this paper, we present the insights provided by a high-resolution, low-cost sensor network deployed across Shanghai Container Terminal 2 (PCT2) and Terminal 4 (PCT4), covering four distinct functional areas to monitor NO and NO_2_ levels. The study period spanned from 11 October to 11 November 2023, including sensor network deployment. The sensor data’s reliability was cross-validated with conventional air quality monitoring stations, and the lowest percentile method was employed to extract regional and local emission contributions, offering detailed insights into the spatiotemporal dynamics of air pollution in the port area. This study aims to identify high-concentration areas and time periods within the port, enabling targeted control measures. By quantifying local emission contributions, we seek to provide evidence-based guidance for policy makers in formulating effective air quality management strategies. Ultimately, our goal is not only to improve air quality within the port but also to contribute to the enhancement of urban air quality by reducing pollutant concentrations in this significant emission source area.

## 2. Methods

### 2.1. Study Area

Shanghai Waigaoqiao Port, located in the Pudong New Area of Shanghai, spans approximately 3.9 square kilometers and includes two primary sectors: Shanghai Container Terminal 2 (PCT2) and Shanghai Container Terminal 4 (PCT4). These terminals are critical pollution sources, with cargo transportation activities leading to emissions of nitrogen oxides (NOx), sulfur dioxide (SO_2_), black carbon, and particulate matter (PM), which are significant pollutants relevant to the port’s operations. To monitor and evaluate air quality control within the cargo terminal, two dedicated air quality monitoring stations (AQMSs) have been established at PCT2 and PCT4, each equipped with Federal Equivalent Method (FEM) pollution analyzers for precise measurements of these pollutants.

**Figure 1:**
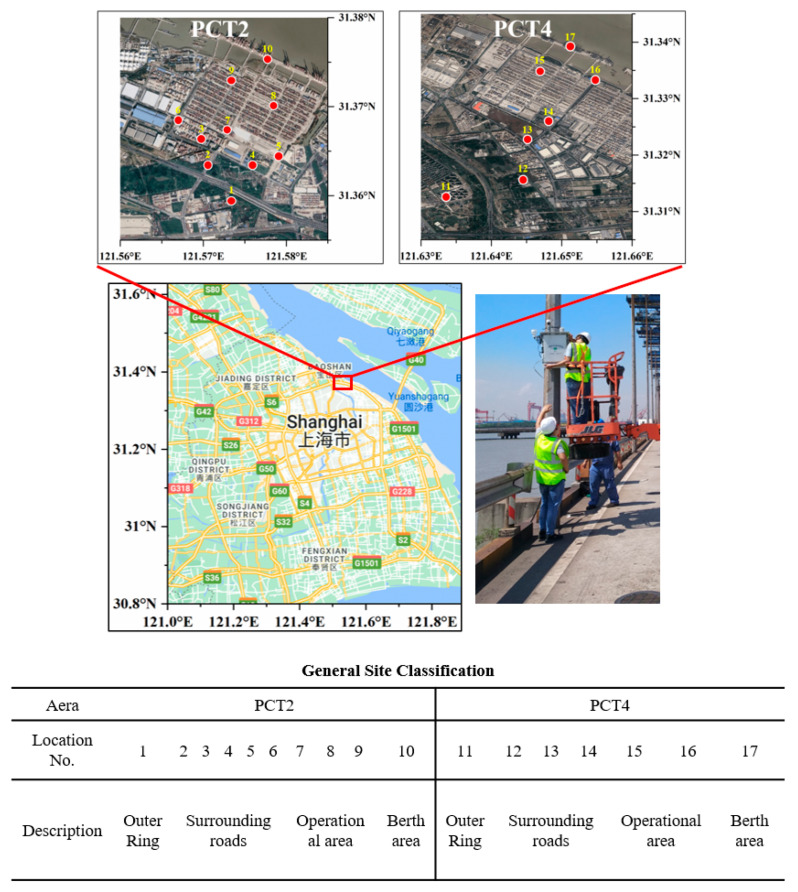
Locations and descriptions of the low-cost sensors deployed in the PCT2 and PCT4.

To thoroughly understand the spatiotemporal characteristics of NO and NO_2_, crucial indicators of port-related air pollution, a supplementary sensor network with a high density of approximately 6–7 sensors per square kilometer was deployed across both terminals. Operating from 21 October to 10 November 2023, this network, detailed in Figure 1, included 17 nodes covering four functional zones: the outer ring (OR), surrounding roads (SRs), operational areas (OAs), and berthing areas (BAs). It should be noted that this study period falls within a single season (autumn), which may be subject to seasonal variations in pollutant concentrations.

- Outer ring (OR): It is the main highway around the city in Shanghai, with more freight vehicles, green belts on both sides of the road, and low building density, providing better diffusion conditions for pollutants.
- Surrounding roads (SRs): The peripheral roads adjacent to the port area, which are the main road for container trucks to enter and exit the port area, are often congested when there is a large number of vehicles and are therefore significantly affected by vehicle emissions.
- Operational areas (OAs): As the hubs of the terminal, these areas are the focus of pollution monitoring due to heavy cargo handling activity and the frequent movement of freight trucks.
- Berthing areas (BAs): These are ship docking areas located on an extension of the terminal, which is also affected by ship emissions in addition to freight vehicles.

The selection of monitoring sites 7 and 16 was strategic, based on their established roles as AQMS locations within the terminal. This facilitated continuous colocation with reference NOx analyzers, providing robust validation data. The sensor network’s strategic deployment allows for precise air quality monitoring across different terminal areas, significantly improving data accuracy and enabling informed actions to mitigate air pollution.

### 2.2. Instrumentation and Data Acquisition

The real-time measurement of NO and NO_2_ concentrations was performed using low-cost sensors integrated into waterproof Mini Air Stations (MAS-AF300, Sapiens, Dongguan, China), whose specifications are listed in Table 1. Each station features modules for NO and NO_2_ measurements with a time resolution of one minute. Independent temperature and humidity sensors within the system monitor and record environmental conditions, ensuring the operational stability of the sensor readings. Data from these stations are transmitted to a cloud platform for real-time monitoring via a 4G network. The sensor nodes are strategically installed on lamp posts throughout the port area. This placement offers several advantages: the sensors are powered by the existing electrical infrastructure of the lamp posts, ensuring a stable and continuous power source; they are mounted at a height of 4 to 5 m above ground level, which helps to capture a representative sample of air quality while minimizing interference from ground-level activities.

The sensors operate on electrochemical principles, with four internal electrodes facilitating the detection process. The target gases, NO and NO_2_, interact with the working electrode (WE), triggering a chemical reduction reaction that alters the current, indicative of gas concentration levels. The sensor technology, grounded in electrochemical detection, incorporates advanced calibration techniques and dynamic baseline tracking [30] to isolate pollution signals from environmental variables. This methodological approach ensures the accurate representation of air quality within the Shanghai Waigaoqiao Port terminals, paving the way for a detailed spatiotemporal analysis of pollution dynamics.

### 2.3. Data QA/QC

The data quality of the sensors is always a concern for users. Given the potential cross-interference of low-cost sensors with temperature, relative humidity, and interfering gases, data quality control and assurance are necessary steps. In this study, three steps are carried out to ensure the reliability of the sensor data.

1. Firstly, the MAS-AF300 integrates an additional gas module with preprocessing capabilities, which employs a polytetrafluoroethylene (Teflon) filter and a Nafion™ tube to balance the humidity of the incoming air and monitor the impact of environmental variables on the gas sensor baseline. Subsequently, algorithms are employed to further compensate for this influence.
2. The second step is field calibration, which was conducted prior to the installation of sensor nodes at the target locations. Field calibration is a widely used method in various studies [31,32,33,34,35]. It entails operating the sensor nodes alongside an FEM instrument in an actual atmospheric environment for a specific duration to monitor pollutants within a targeted concentration range. In our study, the sensor nodes were colocated with a standard monitoring instrument (Model 42i, Thermo Fisher Scientific, Waltham, MA, USA) to simultaneously measure outdoor NO and NO_2_ concentrations, as well as temperature and humidity. The concentration ranges and environmental conditions during the calibration period are crucial for ensuring the validity of the regression model. We typically aim for a maximum pollutant concentration above 50 ppb during the calibration period to ensure the effectiveness of the regression. Additionally, we avoid extreme weather conditions, such as prolonged periods of high relative humidity (above 80%), and maintain a temperature range between 5 °C and 35 °C during calibration. Based on the data collected during field calibration, adjustment coefficients for the sensors are obtained using a multiple linear regression model.
3. The final step is the real-time validation of the sensor data during real-world application. In this study, two sensor nodes were installed at the AQMS located at the cargo terminal, providing us with the opportunity to assess the data quality of the entire sensor network. The baseline of gas sensors tends to drift over time due to their electrochemical characteristics. The validation results of the sensor measurements at monitoring sites 7 and 16 compared to the corresponding measurements from the AQMS serve as the benchmark for the entire sensor network. If a significant number of outliers are observed, field calibration will be repeated to update the calibration coefficients.

To ensure the performance of the sensor, two tiers of data quality objectives are implemented based on the collected data: the coefficient of determination (*R*^2^) and the root mean squared error (RMSE). The Pearson correlation coefficient measures the strength and direction of the linear relationship between the measured values and the reference values. The root mean squared error provides an estimation of the overall deviation between the measured values and the reference values. Lower RMSE values and higher *R*^2^ values indicate a closer agreement between the sensor measurements and the reference measurements, implying higher accuracy. The following Equations (1) and (2) are used for calculations:

(1) RMSE=∑t=1t=N(Sensort−Re⁡ft)2/(N−1) 

(2) R2=1−∑t=1t=N(Sensort−Re⁡ft)2/∑t=1t=N(Sensort−Re⁡f¯)2 

where $Sensor_{t}$ denotes the multi-channel sensor measures at time *t*. Re⁡ft is the reference readings at time *t*, and Re⁡f¯ is the average value of reference readings during the calibration period.

### 2.4. Local Emission Baseline Extraction Method

The measurement of air pollution concentration involves the combination of regional underlying background and local emission [36]. Background levels fluctuate daily due to meteorological changes, whereas local emissions from proximate sources vary more acutely, displaying rapid spikes and high-frequency variations. Distinguishing these components is vital to accurately assess the contribution of local sources to overall pollution levels. In this research, we employed the lowest percentile method with time series analysis to differentiate between background and local emissions in the data obtained from our high-resolution sensor network.

In this study, the lowest percentile method, based on time series analysis, was employed to separate the background signal and local concentration signal from high-temporal-resolution monitoring data, resulting in a time-varying background signal [37]. The analysis involved segmenting the high-resolution data into discrete 8 h periods, a methodologically sound approach reflective of the natural diurnal variation in air quality, rather than human activity patterns. This segmentation is intended to capture the lowest pollution levels within each period, which are less likely to be influenced by localized emission events. These minimum values are then used to establish a time-varying background signal, applying a thin-plate regression spline for smoothing, following the methodologies of Heimann et al. [36] and Shairsingh et al. [37]. The selection of an 8 h window for this analysis is based on its efficacy in capturing the inherent fluctuations in air quality, providing a scientifically grounded method for background level determination. This process ensures that the background concentration reflects broader environmental trends rather than short-term, localized emission spikes, thus allowing for a more accurate isolation and analysis of local emission impacts on air quality [38]. This choice also aligns with common societal rhythms, particularly standard work shifts, which can significantly influence pollution patterns in urban and industrial areas. The 8 h segmentation effectively captures daily cycles of pollutant concentrations, including morning and evening peaks related to traffic and industrial activity, while offering practical computational efficiency for robust statistical analysis of background concentrations in the port environment.

## 3. Results

### 3.1. Comparison During Campaign Period

**Figure 2:**
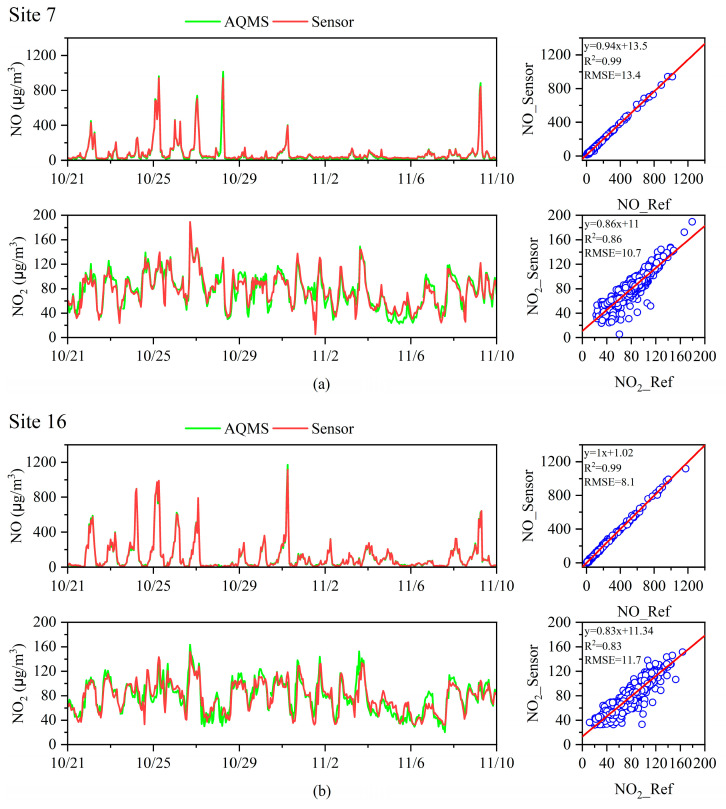
Comparison results of NO and NO_2_ measurements during the campaign period of sensors and reference AQMS at the (**a**) PCT2 (site 7) and (**b**) PCT4 (site 16). For plotting, 1 h average data are used.

In this study, two sensors (site 7 and site 16) were deployed at the local AQMSs in PCT2 and PCT4, respectively. Figure 2 presents a time-series comparison of NO and NO_2_ measurements from these colocated sensors and the AQMS reference data, with sensor data aggregated into 1 h intervals to align with the FEM gas analyzer’s resolution (Model 42i, Thermo Fisher Scientific, Waltham, USA). The sensors demonstrated strong alignment with the reference measurements, capturing large-scale variations effectively, with peaks reaching around 1000 μg/m^3^ for NO and 160 μg/m^3^ for NO_2_.

The correlation analysis yielded impressive *R*^2^ values of 0.99 for NO at both sites, indicating a near-perfect match with the AQMS data. The root mean-square error (RMSE) values (site 7 and 16) for NO were 13.4 μg/m^3^ and 8.1 μg/m^3^, underscoring the sensors’ accuracy, respectively. However, the performance metrics for NO_2_ were slightly lower, with *R*^2^ values of 0.86 and 0.83, reflecting a more complex atmospheric chemistry of NO_2_, which forms through the oxidation of NO and may display more variable concentration levels due to environmental factors and oxidation processes. Additionally, the difference in concentration ranges between NO and NO_2_ may contribute to this discrepancy. NO, as a primary emission, exhibits a wider concentration range (up to 1000 μg/m^3^), while NO_2_, being a secondary pollutant, shows a narrower range (maximum around 200 μg/m^3^), potentially leading to larger relative errors and lower *R*^2^ values in regression analyses for NO_2_.

The validation results from the two colocated sensors at sites 7 and 16 demonstrated high accuracy in measuring NO and NO_2_ concentrations, closely aligning with AQMS reference data. However, extending this level of confidence to the entire network of 17 sensors requires a systematic and comprehensive validation approach. According to recent literature [39,40], validating the precision of individual sensors is crucial, but it is equally important to verify the collective performance and reliability of the entire sensor network. Variations between the sensors’ readings and the AQMS reference data might indicate potential baseline drift or sensor degradation over time.

To address this and ensure consistent data quality across the network, a structured validation strategy was implemented. Each sensor in the network underwent periodic side-by-side comparisons with the AQMS reference instruments, not limited to the initial two sensors. A rigorous calibration protocol was established to complement this strategy. Sensor validation was performed at intervals not exceeding three months, utilizing a systematic rotation method. This approach involved periodically co-locating network sensors with AQMSs for comparative measurements, with each comparison period lasting a minimum of 48 h to ensure comprehensive and reliable calibration data. This ongoing cross-validation process was crucial for monitoring and correcting any deviations or drifts in sensor performance over time. By adopting this comprehensive validation approach, informed by current research and best practices, this study ensured that the entire sensor network maintained high levels of accuracy and reliability.

### 3.2. Temporal Trends

**Figure 3:**
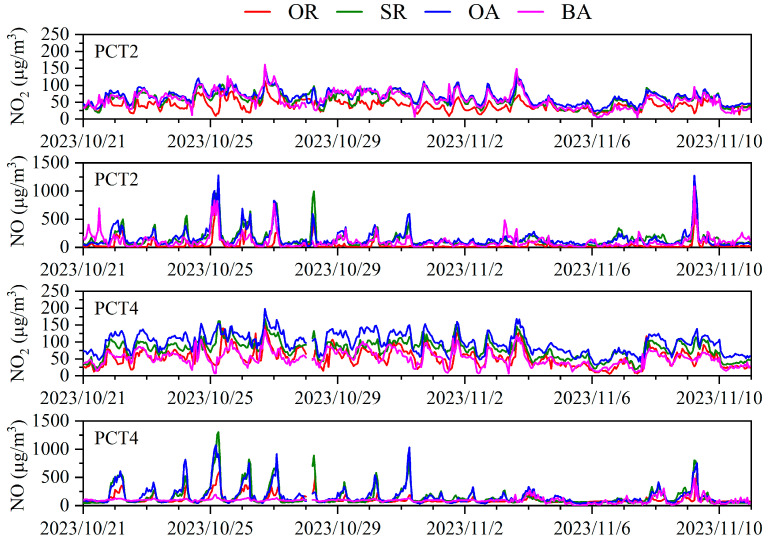
Time series of NO_2_ and NO in four functional zones in PCT2 and PCT4.

Figure 3 presents the time series for NO and NO_2_ concentrations across the four functional zones in PCT2 and PCT4, from 21 October to 10 November 2023. Consistent with the figure, NO levels at PCT2 and PCT4 showed similar fluctuating patterns, with notable peaks in the surrounding roads (SRs) and operational areas (OAs), suggesting these zones are hotspots for emissions, likely due to heavy-duty diesel truck activity and intensive cargo operations. A particularly severe pollution episode occurred between October 25 and 28 October, and on 9 November, with 25 October exhibiting exceptionally high NO values exceeding 1000 µg/m^3^ in the SRs and OAs of both terminals. This indicates the substantial impact of primary emissions from vehicular and ship activities.

NO_2_ concentrations, in contrast, remained relatively uniform in both terminals throughout the study period, without the pronounced spikes characteristic of NO. This pattern reflects NO_2_’s nature as a secondary pollutant predominantly produced from the reaction of NO with ozone. Despite the excess NO emissions, the formation of NO_2_ appears consistent, suggesting a balanced dynamic between NO oxidation and ozone availability. Additionally, the spread of pollution is likely influenced by meteorological conditions such as wind direction and speed, which can disperse emissions across and beyond the terminal areas.

**Figure 4:**
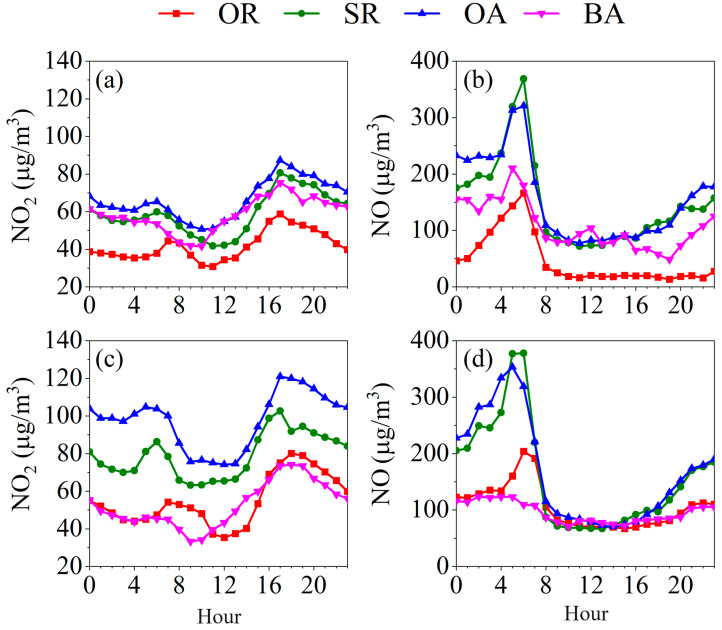
Diurnal patterns of NO_2_ and NO in four functional zones in (**a**,**b**) PCT2 and (**c**,**d**) PCT4.

Figure 4 shows the diurnal variations in NO and NO_2_ in four functional zones in PCT2 and PCT4 during the campaign. Similar diurnal patterns of NO were observed in different functional zones, which showed an increasing trend during the whole nighttime, reached a peak in the early morning at approximately 06:00, and then decreased until mid-daytime. This phenomenon could be related to the local policies on the cargo transportation of heavy-duty diesel trucks and incoming/outgoing cargo vessels, which are only allowed during the nighttime. Furthermore, NO showed similar values in surrounding roads and terminal operating areas, extremely higher than the other two zones, which could be related to the cargo transportation processes and working areas. NO_2_ exhibited slightly different diurnal patterns with NO in four functional zones. NO_2_ showed obviously bimodal patterns and reached peak values at about 06:00 and 17:00. Furthermore, the evening NO_2_ peak values were significantly higher than those observed in the morning, a pattern distinct from that of NO. The lower concentrations during the daytime were mainly related to the photolysis reactions of NO_2_, while the higher concentrations during the nighttime were generally related to accumulation by reactions of NO and O_3_, as well as the lower boundary layer height of the atmosphere. Additionally, it is crucial to consider the influence of coastal meteorology, particularly land and sea breezes, on pollutant concentrations in port areas. These atmospheric circulation patterns contribute to complex temporal variations in pollutant levels, potentially exacerbating nighttime concentrations. The interplay between local emission sources and meteorological factors presents a significant challenge in distinguishing their respective impacts on air quality in coastal port environments.

### 3.3. Spatial Trends

**Figure 5:**
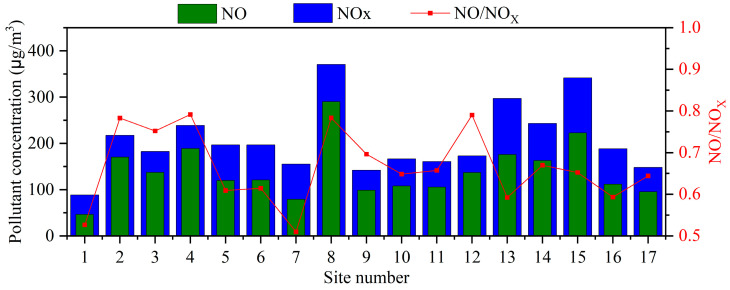
Histogram plots of the average concentrations of NO and NOx at each monitoring site. The red line represents the NO/NOx ratio.

A statistical summary of NO and NO_2_ concentrations recorded at 17 monitoring sites over the data collection period is shown in Table 2. Figure 5 presents the histogram plots of the average concentrations of NO and NO_x_ at each monitoring site together with the NO/NO_x_ ratio. The NOx emitted from mobile sources mainly consist of NO and NO_2_. NO is produced as a result of the reaction between nitrogen and oxygen in the combustion process of automobile engines under high temperature and pressure conditions. NO further reacts with oxygen in the atmosphere to form NO_2._

The average concentration range of NO across the various stations ranges from 47 μg/m^3^ to 289 μg/m^3^, while the average concentration range of NO_2_ is 42 μg/m^3^ to 121 μg/m^3^. It is worth noting that the NO concentrations at all stations are higher than the NO_2_ concentrations, which is contrary to typical atmospheric conditions where NO_2_ concentrations are generally higher than NO concentrations. This is because in the atmosphere, NO reacts with oxygen to form NO_2_, and NO_2_ is relatively stable and less prone to further oxidation into other compounds. The higher NO concentrations compared to NO_2_ concentrations in the terminal area can be attributed to the high concentrations of NO emitted from strong traffic emission sources in that region. This can lead to the inhibition or incomplete oxidation process of nitrogen oxides, resulting in higher NO concentrations relative to NO_2_. Overall, the highest average concentration of NOx was observed at site 8 with a value of 370 μg/m^3^, followed by monitoring sites 15 and 13 with values of 341 μg/m^3^ and 296 μg/m^3^, respectively. The lowest average concentration of NOx was observed at monitoring site 1 with a value of 89 μg/m^3^, followed by monitoring site 7 and 9 with values of 122 μg/m^3^ and 142 μg/m^3^, respectively. This indicates significant spatial heterogeneity in the concentration of NOx at both PCT2 and PCT4. The standard deviation range of NO across the stations is from 66 μg/m^3^ to 252 μg/m^3^, while the standard deviation range of NO_2_ is from 13 μg/m^3^ to 51 μg/m^3^. Meanwhile, monitoring sites with higher average concentrations of NOx are often accompanied by larger standard deviation values, indicating greater variability among the NOx measurements at those stations. The higher concentrations of emissions contribute to the higher NOx levels, resulting in larger fluctuations in the measured values.

The ratio of NO to NOx is often used to assess the degree of oxidation of nitrogen oxides. A lower NO/NOx ratio indicates that nitrogen oxides are less oxidized to nitrogen dioxide by oxygen, which may suggest stronger source emissions or poorer meteorological conditions. The NO/NO_X_ ratios at all monitoring sites are greater than 0.5, indicating that NO contributes to most of the NO_X_ concentration. Generally, monitoring sites with higher NO_X_ concentrations are accompanied by higher NO/NO_X_ ratios. It is worth noting that the highest NO/NO_X_ ratio of 0.8 is observed at monitoring site 12, even though its NO_X_ concentration is not very high. This could be attributed to local dispersion conditions leading to the accumulation of NO in the vicinity. For monitoring site 8, which has the highest NO_X_ concentration, the NO/NO_X_ ratio is 0.8, possibly due to strong local traffic emissions. Additionally, the lowest NO/NO_X_ ratio is observed at monitoring site 7 with a value of 0.5, followed by monitoring site 1 with a value of 0.55. Despite the relatively high NO_X_ concentration at monitoring site 7, it may have favorable gas exchange conditions that promote the oxidation of more NO to NO_2_. On the other hand, monitoring site 1 has a lower NO_X_ concentration and is located farther away from emission sources, resulting in less influence on the NO/NO_X_ ratio.

**Figure 6:**
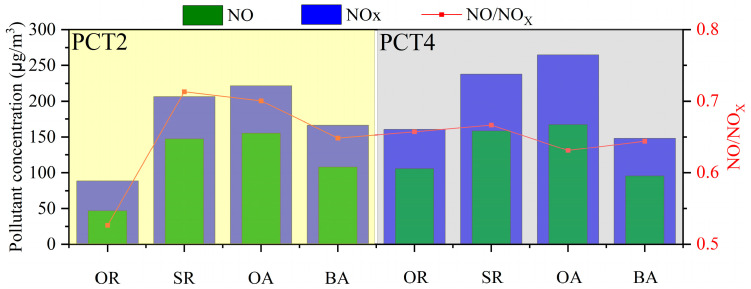
Histogram plots of the average concentrations of NO and NOx in different functional zones. The red line represents the NO/NOx ratio.

Figure 6 shows the average concentration of NO and NO_x_, along with the NO/NOx ratio in four functional zones in PCT2 and PCT4 during the campaign period. PCT2 and PCT4 exhibit a similar pattern, with the average concentrations of NOx in the four functional zones ranked as follows: OA > SR > BA > OR. Consider the following aspects: firstly, the OA is the most active zone within the terminal, involving cargo handling and vessel operations, resulting in higher emissions and consequently the highest concentration of NOx. Secondly, the SRs bear a significant volume of traffic, especially freight vehicles and heavy trucks, which are important sources of NOx emissions from vehicle exhaust. Therefore, the NOx concentration is also higher in the vicinity of these roads. In the BAs, the concentration of NOx is influenced by the exhaust emissions from trucks entering and leaving the berthing area. Lastly, the OR is an area that is further away from the main emission sources and has a more open space, allowing for better air dispersion. Furthermore, for the high-concentration functional areas of the OAs and SRs, the NOx concentration in PCT2 is lower than in PCT4, but the NO/NOx ratio is higher in PCT2. This can be explained by the fact that although the NOx emissions from the OA and SR in PCT2 are lower than in PCT4, the conditions for dispersion are relatively poor, resulting in a lower conversion of NO to NO_2_. It is worth noting that in PCT2, both the average NOx concentration and the NO/NO_x_ ratio in the OR are the lowest.

### 3.4. Local Emission Characteristics

**Figure 7:**
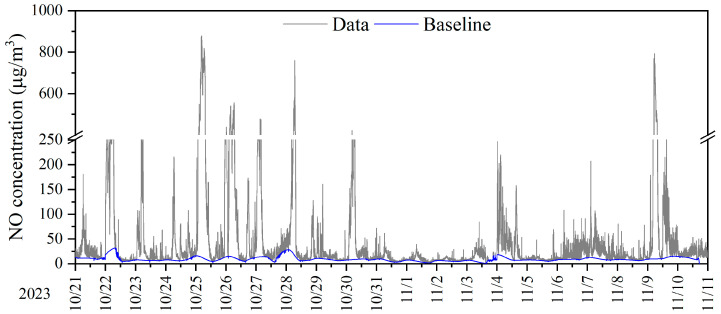
Time series of NO concentration for monitoring site 1. Data recorded at 1 min resolution are shown in grey color, while the regional baseline extracted data using the lowest percentile method are shown in blue color.

Figure 7 illustrates the separation of the background signal from the monitoring data using the lowest percentile method, taking NO measurements at monitoring site 1 as an example. Frequent high spikes were observed in the sensor-collected NO data, with the highest concentrations reaching up to 900 μg/m^3^, which are attributed to emissions from non-continuous mobile emission sources. However, the concentration range of the baseline signal (Figure 7, blue curve) was between 0 and 31 μg/m^3^. Lower NO baseline concentrations were observed during the daytime, possibly due to the influx of cleaner air from the free troposphere, which typically has low NO concentrations, mixing into the boundary layer. In contrast, the stable nighttime boundary layer trapped pollutants near the surface, leading to higher NO concentrations during the night. Additionally, the daytime regulation of diesel trucks in the city was also a contributing factor to the lower baseline signal during the day. This study demonstrated that high-resolution data collected from sensors can identify different regional influences and local contributions without making prior assumptions about the emission inventories of the deployment location.

**Figure 8:**
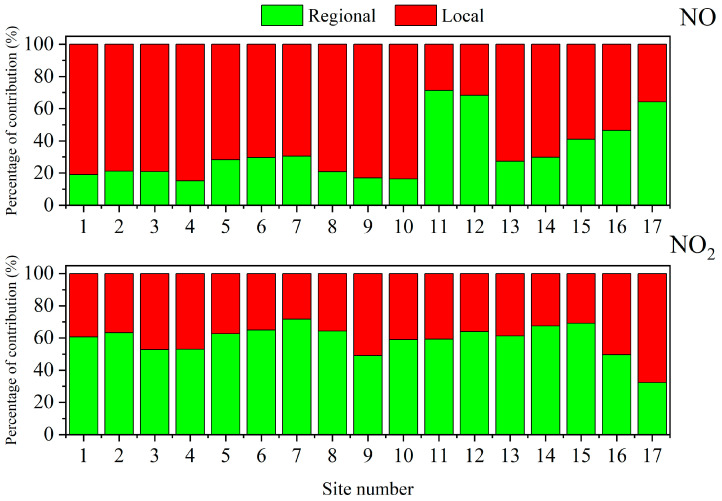
Histogram of percentage contribution of local emission (denoted in red) and regional baseline (denoted in green) for NO and NO_2_.

Figure 8 provides valuable insights into the distribution of regional baseline and local emission contributions within the sensor network located within the cargo terminal. The findings highlight the significant role of local emissions as the primary contributors to NO concentrations, with levels peaking at over 80% in some cases. This emphasizes the importance of addressing local sources of pollution within the terminal environment. However, it is notable that sites 11, 12, and 17 exhibit a different pattern, where the regional baseline becomes the major contributor to NO concentration, accounting for approximately 60% to 70%. These sites’ unique position at the periphery of the terminal, relatively distant from traffic emission sources, plays a crucial role in this disparity.

While these sites do experience higher levels of NO concentration, the majority of the contribution arises from the elevated regional background resulting from the dispersion and mixing of pollutants emitted from various pollution sources. It suggests that the impact of specific pollution hotspots is less significant at these locations compared to the influence of broader regional pollution sources. Furthermore, it is important to consider the relationship between NO and its oxidized product, NO_2_. As mentioned earlier, NO_2_ concentration is the result of the oxidation of NO. Unlike NO, which is directly emitted from traffic sources, the accumulation of NO_2_ concentration occurs gradually over time, accompanied by the regional dispersion of pollutants. This aligns with the expected behavior of NO_2_ as a pollutant that evolves through atmospheric chemical reactions and regional transport. The source apportionment analysis for NO_2_ further confirms the dominance of the regional baseline as the primary contributor. With the exception of monitoring station 17, where other factors may be at play, the contribution of the regional baseline to NO_2_ concentration surpasses 50% at all other stations. This reinforces the significance of understanding and addressing broader regional pollution sources to effectively mitigate NO_2_ levels within the cargo terminal.

## 4. Discussion

Shanghai Waigaoqiao Port, one of the world’s busiest container hubs, contributes substantially to the local economy through its vast container throughput. However, the associated port activities—including container handling, storage, and vehicular movements—pose considerable air quality challenges for the surrounding communities and the broader urban environment. The local authorities have shown significant concern about the emissions from the terminal, leading to the establishment of two regulatory AQMSs within the terminal area to provide highly accurate pollution monitoring. However, due to the spatial variability of pollutants within the terminal, there can be significant differences in pollution concentrations within a few meters’ distance. Additionally, pollution emissions from fuel combustion, especially from mobile traffic sources, often occur at high concentrations within minutes or even seconds. However, the time resolution of the FEM pollutant analyzers employed by the AQMSs is typically 1 h, which is insufficient to capture rapid changes in pollutant concentrations. Therefore, in this study, we deployed 17 additional sensors as a supplement to the AQMSs, distributed across four different functional zones within PCT2 and PCT4, to enhance high spatiotemporal resolution monitoring of NO and NO_2_ within the terminal.

During the campaign period, two sensors were operated in parallel with the AQMS within the terminal. They served to validate the data quality for the entire network. The results indicated a high consistency between the measurements of NO by the sensors and the FEM analyzer, with an *R*^2^ value of 0.99 and RMSE ranging from 8.1 μg/m^3^ to 13.4 μg/m^3^. The validation results for NO_2_ were also acceptable, with an *R*^2^ value ranging from 0.83 to 0.86 and an RMSE ranging from 10.7 μg/m^3^ to 11.7 μg/m^3^.

Overall, compared to urban environments, the terminal exhibits high concentrations of NOx. During the monitoring period, the hourly average concentrations of NO and NO_2_ can reach up to 1000 μg/m^3^ and 180 μg/m^3^, respectively. Additionally, both NO and NO_2_ display distinct diurnal patterns, with concentrations increasing throughout the night and reaching peak values around 06:00, followed by a gradual decrease until mid-day. This phenomenon may be attributed to local policies regarding heavy-duty diesel trucks and shipping vessels for cargo transportation. The sensor network provides a clear spatial view of the distribution of NO and NO_2_ within the terminal, with the highest recorded NOx concentration reaching 370 μg/m^3^ (site 8) and the lowest at 89 μg/m^3^ (site 1), providing valuable insights for environmental management.

The lowest percentile is a useful method for studying variations in time series data. It can extract underlying trends from high-temporal-resolution measurements, enabling the quantification of the contributions of regional background and local emissions to the total pollutant concentrations. The study results highlight the advantages of improving monitoring time resolution in identifying emission contributions. Coupled with the increased spatial density of monitoring provided by the low-cost sensor network, it becomes possible to offer additional information about air pollution patterns and sources, thereby better characterizing the high variability and complexity of pollution.

## Figures and Tables

**Table 1:** Technical and system specifications of MAS-AF300.

Technical Specification					
**Pollutant**	**Monitoring Range**	**Resolution**	**Reading Errors**	**Detection Limit**	**Repeatability**
NO	0–5 ppm	0.1 ppb	±5 ppb or 15% measurement	5 ppb	≤2%
NO_2_	0–5 ppm	0.1 ppb	±5 ppb or 15% measurement	5 ppb	≤2%
**System Specification**					
Dimension			800 × 584 × 253 mm (H × W × D)		
Weight			15 Kg (including battery)		
Operating Temperature			−10 to 50 °C		
Operating Relative Humidity			0 to 99%		
Data Transmission			4G module		
Time Resolution of Data Collection			1 min		
Sampling Flow Rate			0.8 LPM		

Note: NO: 1 ppb = 1.25 μg/m^3^; NO_2_: 1 ppb = 1.91 μg/m^3^ at standard temperature and pressure (20 °C, 1 atm).

**Table 2:** Descriptive statistics of NO and NO_2_ concentrations at different monitoring sites.

Site ID	NO (μg/m^3^)					NO_2_ (μg/m^3^)				
	25th Percentile	Median	Average	75th Percentile	SD	25th Percentile	Median	Average	75th Percentile	SD
1	9.11	14.47	47.36	29.35	104.41	30.75	40.39	41.86	51.87	16.31
2	50.52	102.24	169.68	193.50	212.35	31.57	48.38	47.13	60.27	20.15
3	30.42	67.13	136.97	148.47	207.79	28.29	45.51	45.21	59.66	23.06
4	32.43	97.95	188.85	233.43	252.61	29.11	47.36	49.77	67.45	28.03
5	36.45	65.26	119.58	127.57	161.76	55.35	75.65	76.84	94.51	28.60
6	31.89	76.51	120.64	165.09	130.24	54.74	78.52	76.56	96.97	31.07
7	23.45	34.17	79.07	74.37	130.56	33.62	42.44	42.88	50.43	12.75
8	98.89	194.97	289.78	367.16	293.95	60.27	79.54	80.30	99.02	28.50
9	18.09	45.29	98.75	116.98	160.07	31.16	42.64	43.05	53.51	17.94
10	28.14	61.51	107.89	124.89	156.11	38.13	58.84	58.54	75.44	27.14
11	69.81	75.98	105.56	96.48	80.39	33.83	53.10	55.05	71.96	27.51
12	90.72	96.08	136.40	119.66	104.10	26.45	34.65	36.31	44.08	13.89
13	38.86	81.07	175.83	216.04	221.01	83.03	122.80	121.08	155.80	51.04
14	39.93	71.42	162.58	168.04	242.09	56.99	81.18	80.29	100.66	31.28
15	99.43	153.83	222.55	266.49	194.26	86.51	119.11	118.79	147.60	41.04
16	76.78	99.83	170.71	196.04	172.43	29.73	56.58	54.93	77.49	30.85
17	71.02	93.00	95.46	106.93	66.49	33.42	52.07	52.74	69.70	26.93

## Data Availability

The data presented in this study are available upon request from the corresponding author.
